# A Case Report of Inhaled Nitric Oxide for Transfusion-Related Acute Lung Injury

**DOI:** 10.7759/cureus.41552

**Published:** 2023-07-08

**Authors:** Satoshi Kometani, Ryo Misawa, Michihiko Kawai, Hiroshi Seki, Mimiko Tabata

**Affiliations:** 1 Anesthesiology, Yamato Seiwa Hospital, Yamato, JPN; 2 Cardiovascular Surgery, Yamato Seiwa Hospital, Yamato, JPN

**Keywords:** venovenous extracorporeal membrane oxygenation, inhaled nitric oxide, blood transfusion safety, acute respiratory distress syndrome [ards], transfusion-related acute lung injury

## Abstract

Transfusion-related acute lung injury (TRALI) is an acute respiratory distress syndrome (ARDS) occurring during or within six hours after transfusion. On the other hand, while inhaled nitric oxide (iNO) temporarily improves arterial oxygenation with selective pulmonary vasodilation, there is no evidence of mortality reduction in ARDS. We herein report a case in which TRALI was diagnosed with severe hypoxemia during cardiovascular surgery, and extracorporeal membrane oxygenation (ECMO) was avoided by using iNO for respiratory management. Administering iNO to patients with acute respiratory failure may be useful as a bridging therapy to help patients recover. However, further evidence is needed before this treatment can become standard practise.

## Introduction

Transfusion-related acute lung injury (TRALI) is a type of acute respiratory distress syndrome (ARDS) occurring during or within six hours after transfusion [1]. It has a mortality rate of 5-25% and is the most severe nonhemolytic transfusion reaction [2]. However, the incidence is infrequent at 1:130,000 blood products [3]. TRALI is also challenging to identify because most ARDS cases are due to infectious diseases, so they are often underdiagnosed and underreported, especially in critically ill patients [4]. Therefore, the clinical discussion is insufficient, no specific treatment has been established, and the primary strategy is discontinuing transfusions and providing supportive therapy for ARDS.

On the other hand, inhaled nitric oxide (iNO) is a selective pulmonary vasodilator that does not cause systemic effects [5,6]. However, while iNO has been reported to reduce the need for extracorporeal membrane oxygenation (ECMO) in the persistent pulmonary hypertension of newborns [5], there is no evidence of its reductive influence on mortality or length of stay in the intensive care unit (ICU) or hospital in adult ARDS patients [6]. For the above reasons, the U.S. Food and Drug Administration has approved iNO only for treating hypoxic neonates with pulmonary hypertension. Nevertheless, iNO is also commonly used as a pulmonary vasodilator in adults [7]. In Japan, administering iNO to treat adult pulmonary hypertension during the perioperative period of cardiovascular surgery was approved in 2015 and is now covered by public insurance.

We experienced a case in which TRALI was diagnosed with severe hypoxemia during cardiovascular surgery, and ECMO was avoided by using iNO for respiratory management. In this report, we will discuss the critical points of the diagnostic process and the usefulness of iNO in treatment.

Written informed consent was obtained from the patient for the publication of this report. This article adheres to the CAse REports (CARE) guidelines.

## Case presentation

A 71-year-old man was presented who was 164 cm tall, weighed 53 kg, and had a body mass index of 19.7 kg/m^2^ (American Society of Anesthesiologists Physical Status II). He was referred to our hospital to be treated for a saccular aneurysm at the distal aortic arch with a horizontal diameter of 46 mm. The aneurysm morphology indicated a high risk of rupture, so a total aortic arch replacement was scheduled. The patient's medical history included an open abdominal aortic aneurysm repair two years prior to this presentation. He also had a history of hypertension, dyslipidemia, and type 2 diabetes and was well-medicated with azilsartan, rosuvastatin, sitagliptin, and metformin. No abnormal findings were identified in preoperative electrocardiograms, chest X-rays, respiratory function tests, echocardiography, or blood tests; therefore, we deemed surgery to carry a low risk for this patient.

General anesthesia was introduced using midazolam, remifentanil, and rocuronium, with maintenance performed with sevoflurane, propofol, remifentanil, and rocuronium. A central venous catheter and pulmonary artery catheter were implanted from the right internal jugular vein, followed by transesophageal echocardiography, which confirmed that the left ventricular ejection fraction was 54% with no regional wall motion abnormalities. The total aortic arch replacement was performed under deep hypothermic circulatory arrest, and four units of packed red blood cells and four units of fresh frozen plasma were transfused during cardiopulmonary bypass.

Shortly after the cessation of cardiopulmonary bypass, we transfused four additional units of fresh frozen plasma to correct hypofibrinogenemia. Thirty minutes after transfusion, the PaO_2_/FiO_2_ ratio was low at 171 (pressure-controlled ventilation [PCV]: positive end-expiratory pressure [PEEP] 5 cmH_2_O), indicating moderate hypoxemia based on the Berlin criteria for ARDS [8]. Regarding mechanical ventilation parameters, tidal volume decreased, showing a deterioration of lung compliance. Transesophageal echocardiography and the pulmonary artery catheter revealed no findings suggesting an increase in left atrial pressure or excess body fluid causing hypoxemia, and the pulmonary capillary wedge pressure (PCWP) was 8 mmHg, so the condition was considered noncardiogenic and determined to be ARDS. The trigger for ARDS was regarded as TRALI type I using the criteria redefined in 2019 [9].

Since there was no pulmonary hypertension at that point, iNO could not be performed, as it is not approved for use under such conditions by the Japanese insurance system. Based on the above circumstances, we discontinued transfusions and performed an alveolar recruitment maneuver and lung-protective ventilation with high PEEP (PCV: PEEP 8 cmH_2_O), and surgery was subsequently completed upon confirming surgical hemostasis. The operation time was four hours and five minutes, the anesthesia time was five hours and 20 minutes, the cardiopulmonary bypass time was two hours and 35 minutes, the deep hypothermic circulatory arrest time was 46 minutes, the intraoperative fluid balance was +5,060 ml, and the transfusion amount was 2,480 ml (packed red blood cells were 560 ml and fresh frozen plasma was 1,920 ml).

**Figure 1:**
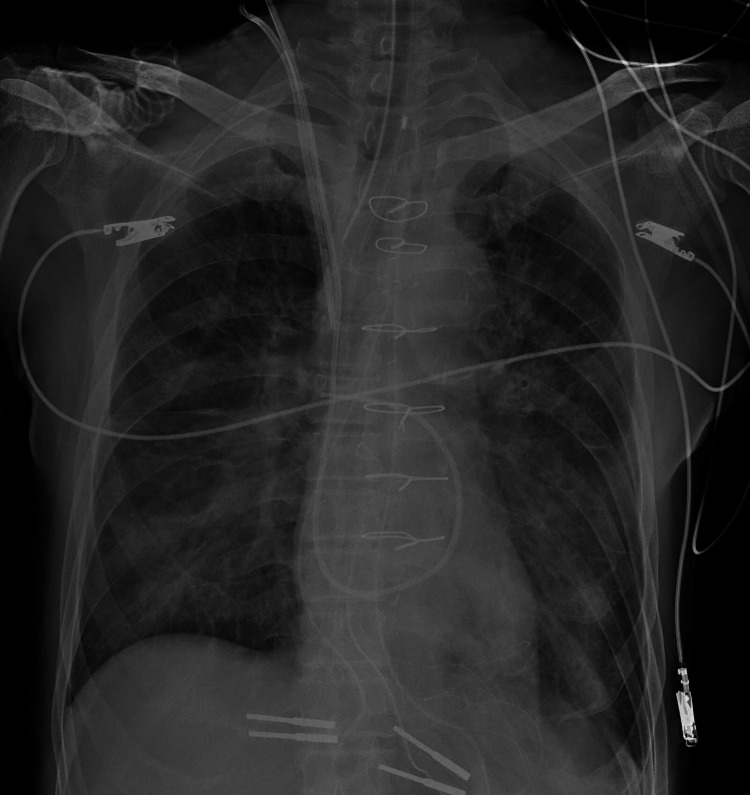
Chest X-ray at the onset of severe hypoxemia in the intensive care unit

**Figure 2:**
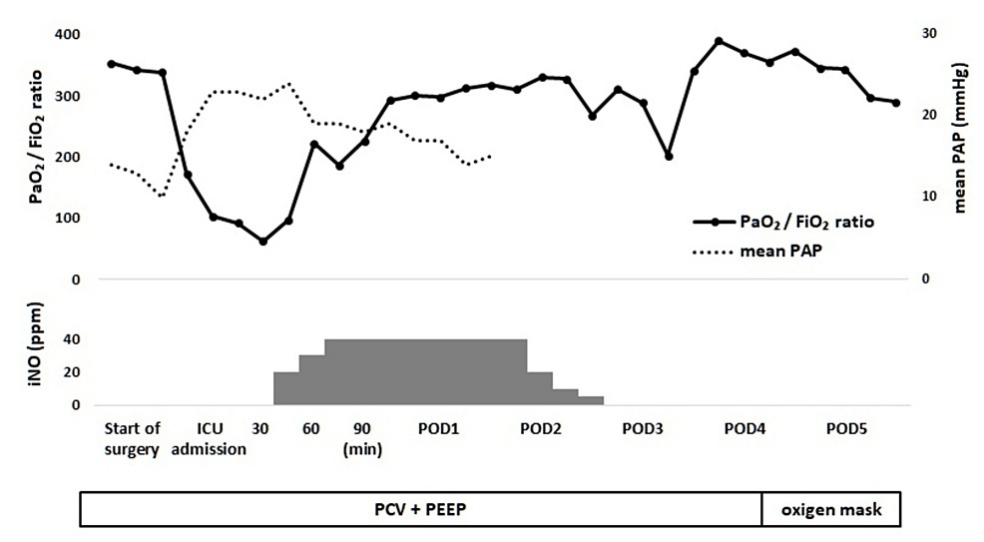
Perioperative time course regarding transfusion-related acute lung injury and inhaled nitric oxide PAP: pulmonary artery pressure; iNO: inhaled nitric oxide; ICU: intensive care unit; POD: postoperative day; PCV: pressure-controlled ventilation; PEEP: positive end-expiratory pressure

However, further deterioration was observed on admission to the ICU, with a mean pulmonary artery pressure rising to over 20 mmHg and a PaO_2_/FiO_2_ ratio of 103. The PCWP was not elevated, and transesophageal echocardiography showed no new left ventricular wall motion abnormalities or mitral insufficiency. In addition, the NT-proBNP value was within the normal range at 13 pg/ml. Based on the above, we considered the patient to have pre-capillary pulmonary hypertension, which falls under group 3 based on the clinical classification of pulmonary hypertension [10]. Although an alveolar recruitment maneuver was again immediately performed, no improvement was seen. After 30 minutes, a yellow, foam-like, serous secretion erupted from the endotracheal tube. The secretions were suppressed to some extent by increasing the PEEP to 15 cmH_2_O, but the patient experienced severe hypoxemia with a PaO_2_/FiO_2_ ratio of 62. In addition, bilateral pulmonary infiltrates accompanied by air bronchograms were observed on chest X-rays (Figure 1). Veno-venous ECMO was considered for respiratory management; however, the right internal jugular vein was catheterized, and a bicaval dual-lumen ECMO cannula had yet to be introduced at the facility. Therefore, despite the limited evidence for ARDS, we decided to administer iNO to improve pulmonary hypertension and oxygenation to the extent allowed by the Japanese insurance system. iNO was started at 20 ppm and maintained at 20 to 40 ppm using INOFLOW™ (Mallinckrodt Pharmaceuticals, Dublin, Ireland). Following the introduction of iNO, the mean pulmonary artery pressure rapidly decreased to under 20 mmHg, and the PaO_2_/FiO_2_ ratio rose to 221 (PEEP 10 cmH_2_O). As a result, we decided not to introduce ECMO. After confirming decreased intratracheal discharge, iNO was discontinued on the second postoperative day. Subsequently, the patient was weaned from mechanical ventilation on the fourth postoperative day and left the ICU on the sixth postoperative day (Figure 2).

This case was later reported to the Japanese Red Cross Society and evaluated as TRALI type I. The institute investigated TRALI-associated leukocyte antibodies, wherein human leukocyte antigen (HLA) class I/class II antibodies were detected from one product of fresh frozen plasma used after the patient was weaned from cardiopulmonary bypass. Subsequently, deoxyribonucleic acid (DNA) typing was performed with the patient's consent, and it was found that the HLA antibodies from the blood product could react with HLA-B62, DR9, DR53, DQ8, and DPw5 of the patient. Finally, a cross-matching test using flow cytometry was carried out on the patient and the product, with a response observed in the patient's B lymphocytes.

## Discussion

We encountered a patient who developed TRALI during cardiovascular surgery, leading to severe hypoxemia in the ICU. Oxygenation disorders in cardiovascular surgery are common, making it difficult for physicians to suspect TRALI immediately. In this case, a certain degree of oxygenation dysfunction was also assumed as a general course of action. However, we believed it was atypical to become sufficiently severe to consider the introduction of ECMO, so this was an opportunity to cite TRALI as a differential diagnosis. ARDS is rare in cardiac surgeries, having been reported in only approximately 1% of cases, even when including emergency surgeries and preoperative high-risk groups [11]. When ARDS develops in a patient with low-risk factors, it is necessary to suspect a transfusion origin.

The pathology of TRALI has been described as a two-hit model. The first hit is the patient's underlying disease contributing to neutrophil priming, and the second is endobronchial vascular endothelium dysfunction due to neutrophil activation associated with transfusion [12]. TRALI is also classified as antibody-mediated/non-antibody-mediated by the second hit [13]. In a retrospective analysis of 49 cases of TRALI, HLA antibodies in donors were confirmed in 73% of them, with proven incompatibility in 48% of the investigated cases [14]. Our case has confirmed the response in cross-matching tests after antibody detection and DNA typing, suggesting that it might have been antibody-mediated.

As mentioned above, TRALI is a clinical diagnosis, so the presence or absence of antibodies does not affect the diagnosis. However, the results affect the blood product management of the donors involved. In this case, under the Japanese Red Cross Society's policy, subsequent donations from the implicated donor will only be used as source materials for plasma-derived medicinal products.

In the respiratory management of TRALI, we believed oxygenation improved significantly with iNO, which helped avoid the need for ECMO and improved the prognosis. iNO contributes to arterial oxygenation by improving the ventilation-perfusion ratio and the intrapulmonary shunt from selective pulmonary vasodilation [15]. Based on this theory, clinical trials have been widely conducted concerning the use of iNO for ARDS; however, those trials did not note an improved survival rate, despite temporary improvements in arterial oxygenation [6]. Since these randomized controlled trials were performed before the widespread adoption of lung-protective ventilation, further investigations are needed. In addition, when discussing the usefulness of iNO for TRALI, the causes of ARDS in past randomized controlled trials were comprehensive, including sepsis, pneumonia, aspiration, toxic gas inhalation, and acute pancreatitis. TRALI is reversible and rapidly improves within 48 hours in most cases [16]. Therefore, short-term bridging therapy was required for respiratory management in the present case. ECMO is also appropriate, but iNO may be more suitable, considering its lower invasiveness. In addition, avoiding ECMO reduces the risk of bleeding, stroke, and embolism, which is especially important for patients after cardiovascular surgery. Similarly, it has been reported that iNO has been successful in treating reexpansion pulmonary edema, which is said to naturally recover within 72 hours in many cases [17]. iNO in acute respiratory failure may improve prognoses depending on the patient's conditions, so further evidence is needed.

## Conclusions

This case involved a patient with TRALI who underwent iNO to improve respiratory management. TRALI is a type of ARDS that is reversible and can rapidly improve when treated appropriately. iNO may be useful as a bridging therapy to help patients recover. However, we must further evaluate its usefulness during the perioperative period and also for its use in the field of critical care.
